# Comparing the mental health of rural-to-urban migrant children and their counterparts in china: Protocol for a systematic review and meta-analysis

**DOI:** 10.1097/MD.0000000000010597

**Published:** 2018-04-27

**Authors:** Jun-hua Zhang, Li-xia Yan, Yang Yuan

**Affiliations:** a School of Education, Jiangsu Key Laboratory for Big Data of Psychology and Cognitive Science, Yancheng Teachers University, Yancheng, China; b Department of Psychology, University of Southampton, Southampton, UK; c School of Education, Soochow University, Soochow; d Department of Pediatrics, Yancheng traditional Chinese medicine hospital, Yancheng, China.

**Keywords:** China, mental health, meta-analysis, migrant children, systematic review

## Abstract

**Background:**

In recent years, the issue of migrant children with peasant parents working in cities has attracted widespread attention in recent years because of the sheer number and the benefits bundled in China's household. The focus has gradually extended from early education opportunities to all aspects of physical and mental development, especially the social adaptation and mental health of migrant children. The negative impact of environment changes on migrant children’ mental health is very worrying for parents and the society. Some studies have found that immigrant children's mental health is significantly lower than their peers, but there are also studies that hold the opposite view. Thus, the mental health status of migrant children is still a controversial issue, which may have a certain relationship with the potential differences in the specific problems of mental health, regions, comparison objects, and researchers. The objective of this protocol is to investigate whether mental health and subdimensions differ between rural-to-urban migrant children and their counterparts living in China and examine study characteristics that might result in differences among studies.

**Methods:**

We will search PubMed, Embase, OVID, ERIC, Web of Science, and Chinese databases including CNKI, Chongqing VIP, and Wan Fang data from start to April 2018. Cross-sectional studies with a comparison of migrant children and their counterparts will be included. The primary outcome will be the mean and standard deviation of mental health and its sub-dimensions. Standardized mean difference is used as the main effect value. Subgroup analyses will be carried out by the location of studies and school type of. Sensitivity analyses will be conducted to assess the robustness of the findings. Analyses will be performed with RevMan and Stata software.

**Results:**

This systematic review and meta-analysis will compare the mental health status of rural-to-urban migrant children and their counterparts living in China.

**Conclusion:**

The results of this systematic and meta-analysis will be helpful to get a more reliable understanding of the mental health of rural-to-urban migrant children and the reasons for the controversy on this issue.

## 1 Introduction

In recent years, the issue of migrant children with peasant parents working in China has attracted widespread attention due to the sheer number of migrant children and provision of public services bundled in hookup policy. Nowadays China government has paid increasing attention to the education issue of migrant children, and it will trigger more and more migrant children to go to cities. Wu et al^[1]^ found migrant children in cities have grown. After acquiring the right to education in cities, the mental health of migrant children has become increasingly prominent. This not only concerns the well-being of migrant, but also the harmony and stability of the entire society and the future of the country.

Every child should have a fair chance of living. However, migrant children become a vulnerable group because migration can be a very stress-inducing phenomenon.^[2]^ The change of living and learning environment can easily lead to psychological problems and social adjustment problems.^[3,4]^ In China, there are huge differences and conflicts between towns and villages in terms of the geographical environment, social conditions, economic level, cultural education, and medical and health care. From rural areas to cities, migrant children need to complete social tasks in addition to adapting to changes in the social environment.^[5]^ Although the initial purpose of every rural-to-urban parents to bring their children to cities is to improve their physical and mental development, environmental changes and lack of parents’ regulatory often threat migrant children's mental health.

There is widespread concern that migration may have adverse effects on children's mental health, which is supported by some studies. The mental health situation of migrant children was found obviously inferior to other Chinese children.^[6]^ Frequent transfer breaks the continuity of the students’ previous learning. The original foundation and poor family conditions make migrant children's preparation for learning lower. Chen et al^[7]^ found that even if compared with non-village children, the migrant children's learning adaptation score is still low. The special living and educational environment of migrant children are prone to affect their psychological health, leading to poor academics, behavioral problems, and personality malformations. Migrant children may have greater difficulties with separation from family, disruption of education, and adaptation to new environments, and they are at greatest risk for having limited access to justice, education, and healthcare.^[8]^ Migrant children are at risk for mental health problems because of environmental changes. Environment changes also have disadvantages in displaying special features, dressing up, and group integration in comparison with their surrounding peers. Although they have strong communicative aspirations, migrant children are not confident enough when dealing with their peers. The survey found that the level of self-concept of migrant children was significantly lower than that of ordinary children in the same age group, showing a strong sense of alienation and loneliness. Migrants face higher rates of physical and mental illness compared with host populations because of a multitude of stressors experienced before, during and after migration. It could not integrate well into the mainstream of the society, and even some serious psychological biases and anti-social tendencies emerged. Therefore, in dealing with teachers, they will deliberately maintain a certain sense of distance; in their associations with their peers, they are often not confident enough.^[9]^ Migrant children lacked a sense of belonging to the city, and self-evaluation was uneven with the perception of discrimination.^[10]^ As the current situation of migrant children's mental health is not optimistic, many scholars have put forward many valuable suggestions from the social, family, and school levels, and clearly pointed out that it is necessary to prioritize the needs of children at all links and levels.^[11]^

In contrast, some other studies found no differences between migrant children and their counterparts. Nicole et al surveyed 838 urban students and 482 migrant students in Guangzhou and found migrant children's psychological, although insignificant, was better than that of urban students.^[12]^ Wang et al found psychological well-being of migration children was better compared to left-behind children.^[13]^ Tang^[14]^ randomly surveyed 100 migrant children from 1 migrant children's school and 1 public school in Shenzhen and found that migrant children have a better mental health. In a study by Li et al,^[15]^ migrant children in preschools with a high rural migrant composition displayed less behavioral problems. Whether the region, age, or school type are moderated factors that lead to the conflicting results is still an important issue.

Other conflicts still exist among the different migrant children. Ni et al found that the identity integration and subjective well-being of migrant children in public schools were higher than those in migrant-exclusive schools. In contrast, Sun et al^[8]^ did a meta-analysis and migrant children in public schools were found to have greater mental health problems than their urban-born counterparts, but those in migrant schools do not have these differences. Therefore, the mental health status of migrant children varies.

To give a relatively comprehensive understanding, the researchers conducted some meta-analysis on this issue. Jin et al reviewed the health of China's rural-urban migrants from 2000 to 2012 and found mental health is an emerging public health priority related to migration.^[16]^ Sun et al^[8]^ found academic performance, social relationships, and discrimination was related to lower the mental health of migrant children. These meta-analyses also had some shortcomings. First, some Chinese database including Chongqing VPN dataset and Wan Fang Dataset, which may have many studies on this subject, were not searched. Second, Sun et al used dichotomous outcomes odds ratio rather than continuous outcomes, and this way will lose some useful information and reduced the accuracy of the finding to a certain extent.^[17]^ Third, mental health is very complex and needs to be compared from multiple dimensions using high-quality scales. Therefore, it is necessary to make a comprehensive evaluation of the mental health status of migrant children and provide a reference for the development of mental health education. Therefore, the objective of this study is to compare the mental health of rural-to-urban migrant children and their counterparts including urban children, left-behind children, and rural children in China. We use the term “counterpart" to include rural, urban, and left-behind children.

## 2 Objectives

The objective of this study is to compare the mental health and its sub-dimensions of migrant children and their counterparts.

## 3 Methods

### 3.1 Eligibility Criteria

#### 3.1.1 Populations

Migrant population under the age of 18 years who have left their Hukou registration place for 6 months or longer with parents from rural areas to cities.

#### 3.1.2 Intervention(s)

Experience of immigration.

#### 3.1.3 Comparator(s)/control

Counterparts without the experience of immigration.

#### 3.1.4 Types of outcome(s)

Mental health scores and its sub-dimensions measured by scales and inventories such as symptom checklist 90, Mental Health Test, Self-rated Scales for Anxiety ,Self-rated Scales for Depression, Positive and Negative Affect Scale for Children (PANAS-C), and other high-quality scales.

#### 3.1.5 Types of Study

This study will include cross-section or observational study. Studies in mental health hospital and clinics will not be included, as these represent a subsample of severe disorders, whereas our meta-analysis focuses on the association between migration and any degree of mental health problems.

### 3.2 Search strategy

We will include published or unpublished studies pertinent to our criteria. Two members will conduct an electronic literature search. The following search resources will be considered: Chinese databases including China National Knowledge Infrastructure, Chongqing VPAN data, and Wan Fang data, PubMed, Embase, OVID, ERIC, and Web of Science (including Science Citation Index Expanded).

Embase search strategy

(Adolescent∗ OR Child∗ OR Teen∗ OR student∗).ti,ab.

(migrant OR floating OR migration OR migrate).ti,ab.

(China∗ OR Chinese).ti,ab.

(mental health∗ OR depression OR anxiety OR lonel∗).ti, ab.

1 AND 2 AND 3 AND 4

### 3.3 Data collecting

#### 3.3.1 Data selection

Endnote software will be used to manage the references with citation, titles, and abstracts and duplicate references will be excluded automated. Literature selection is divided into 2 separate stages:

1. Two authors will independently screen title and abstracts of all nonduplicated articles and will exclude those not pertinent to the criterion. A final list will be agreed with discrepancies resolved by consensus between the two authors. When consensus is not reached, a third senior author will act as arbitrator. Any articles with disagreement between reviewers will be left to the next stage.

2. The full-text version of the articles passing stage 1 screening will be downloaded and assessed for eligibility by two authors, independently. The appendix will provide a complete list of articles excluded at the full-text screening stage, with reasons for exclusion. Discrepancies will be resolved by consensus between the 2 authors and, if needed, a third senior author will act as arbitrator. Data from multiple reports of the same study will be linked together. Where required, we will contact the corresponding author to inquire about study eligibility.

#### 3.3.2 Data extraction

One reviewer will input outcome data from studies included in previous systematic reviews into Excel. This will be independently cross-checked by another reviewer. The following data will be collected from each included study:

- Study details: study citation, year(s) of study or publication, location (city or province, according to the level of economic development, it can be divided into western, central and eastern regions), setting, funding/sponsor (industry or academic);
- Participants details, including number, sex distribution, mean and range of age, and school type in both groups;
- Outcomes: M (mean), N (number of subjects), SD (standard deviation) in both groups. Continuous data will be pooled with a standardized mean difference (SMD) and Hedges’ adjusted g.^[18]^

#### 3.3.3 Risk of bias (quality) assessment

Cochrane Collaboration risk of bias tool will be used by 2 reviewers independently as a reference to assess the risk of bias for each study included.^[19]^ There are 5 bases, including selection bias, performance bias, detection bias, attrition bias, and other bias. The discussion will behold when any disagreement comes out. If necessary, reviewers will Email the authors of the studies for further information.

### 3.4 Data analysis

#### 3.4.1 Data synthesis

Meta-analysis will be conducted using Review Manager and Stata. The migrant children were regarded as the experimental group and the counterparts group as the control group. Using the Der Simonian method and the Laird random-effects model, the pooled estimates of standardized mean difference with 95% confidence intervals will be calculated for the continuous outcomes. *I*^*2*^ and *Q*-statistic test will be used to assess the heterogeneity and variation in pooled estimation.^[20]^ If the data are good enough, then network meta-analysis is further used to compare the direct and indirect 4 groups.

#### 3.4.2 Subgroup analysis

Where significant heterogeneity observed and sufficient data are available, we plan to perform the following subgroup analysis for the primary outcomes according to the following factors: location of migrant children; school type of migrant children; type of the counterparts: counterpart may be urban children, left-behind children, normal rural children; quality of mental health scales; age of children and adolescent; length of the migrant.^[21]^

#### 3.4.3 Funnel plot analysis and meta-regression analysis

Funnel plot analysis will be used to check for publication bias. In addition, in order to investigate the effect of sponsorship, year published, province of migrant children, quality of scales, and type of counterparts on outcome estimate, meta-regression analyses will be conducted.^[22]^

#### 3.4.4 Sensitivity analyses

Sensitivity analyses will be carried out by excluding studies with low-quality mental health scales.

## 4 Conclusion

To know the status of migrant children's mental health is very important for offering mental health service to them. As there are no consistency conclusions, we plan to undertake the present study to increase power to detect an overall treatment effect and identify study characteristics associated with results. This systematic review focuses on the compare of mental health between migrant children and their counterparts (urban children, left-behind children, and normal rural children) in China and wants to find some characteristics leading to this inconsistency. We will make good use of continuous scores of mental health and its sub-dimensions, not just the rate of mental health. Subgroup analysis may find the difference between migrant health and rural, urban, and left-behind children. The region of migrant children and school type maybe an important factor because the government favored a lot of policy to some areas. This may deepen our understanding of migrant children. However, the limitation is primary outcome assessed using many mental health scales. It may increase heterogeneity.

## Author contributions

**Data curation:** Yang Yuan.

**Methodology and Investigation:** Lixia Yan,Yang Yuan.

**Writing – original draft:** Junhua Zhang.

**Writing – review & editing:** Junhua Zhang, Lixia Yan, Yang Yuan.
